# Association Between Co-occurring Anxiety and Depressive Symptoms at Baseline and Risk for Sports-Related Concussion in Collegiate Athletes

**DOI:** 10.1177/23259671241255932

**Published:** 2024-06-19

**Authors:** Garrett A. Thomas, Megan L. Bradson, Kaitlin E. Riegler, McKenna S. Sakamoto, Peter A. Arnett

**Affiliations:** † Department of Psychology, The Pennsylvania State University, University Park, Pennsylvania, USA; ‡ Princeton Neuropsychology-Sports Concussion Center of New Jersey, Princeton, New Jersey, USA; Investigation performed at The Pennsylvania State University, University Park, Pennsylvania, USA

**Keywords:** anxiety, athletic training, depression, head injuries/concussion, mild traumatic brain injury, physiological aspects of sport

## Abstract

**Background:**

There is limited research examining whether mental health problems increase the risk for future concussions, even though these problems are highly prevalent in college-aged populations—including student-athletes.

**Purpose/Hypothesis:**

To examine whether affective disturbance (ie, depressive and anxiety symptoms) at baseline increases the risk for prospective concussion. It was hypothesized that athletes with co-occurring depressive/anxiety symptoms would incur the greatest risk for injury.

**Methods:**

A total of 878 collegiate athletes completed baseline neuropsychological testing. Athletes were separated into the following 4 groups based on self-reported anxiety and depressive symptoms at baseline: healthy controls; depressive symptoms alone; anxiety symptoms alone; and co-occurring depressive and anxiety symptoms. Of the 878 athletes, 88 sustained future concussions. Logistic regression was conducted with prospective concussion (yes/no) as the outcome and the affective group as the predictor. Sport was included as a covariate.

**Results:**

After controlling for sport, athletes in the co-occurring depressive/anxiety symptoms group were more than twice as likely to be diagnosed with a future concussion compared with healthy controls (odds ratio, 2.72 [95% CI, 1.33-5.57]; *P* = .01). The co-occurring depressive/anxiety symptoms group also showed an increased risk for prospective concussion compared with the depressive symptoms alone and anxiety symptoms alone groups, respectively. However, the results were not statistically significant. Athletes in the depressive symptoms alone and anxiety symptoms alone groups did not show a significantly increased risk for prospective concussion compared with healthy controls.

**Conclusion:**

Athletes with co-occurring depressive/anxiety symptoms at baseline showed a notably increased risk of being diagnosed with a future concussion, even after controlling for sport. This suggests that co-occurring depressive/anxiety symptoms infer a unique risk that is associated with a greater susceptibility to concussion diagnosis.

Nearly 20% of young adults between the ages of 18 and 25 years experience some form of mental health difficulty, such as depression or anxiety.^20^ A recent National College Health Association executive summary of nearly 68,000 college students across 98 universities and colleges found that most respondents (58.7%) reported experiencing “more than average stress” or “tremendous stress.”^1^ Further, a substantial number of students reported being diagnosed with or treated for anxiety (24.3%), depression (20%), or co-occurring anxiety/depression (16.5%) within the last year.^1^ Importantly, collegiate student-athletes are not impervious to mental health difficulties. While there are numerous benefits associated with athletics and some research has suggested that participation in sports is a protective factor against mental health difficulties,^2^ other research has found that student-athletes are just as likely as their nonathlete peers to experience issues related to mental health.^27^ Further, a systematic review of depression in collegiate athletes conducted by Wolanin et al^37^ found that depression in athletes is prevalent and certain subpopulations of collegiate athletes may show higher rates of depression compared with nonathletes. Relatedly, student-athletes are subject to unique stressors that may increase their risk for mental health difficulties—for example, extensive time demands, pressure to perform athletically and academically, pressure to maintain scholarship, internal and external pressure to win, and increased visibility related to their student-athlete status. Student-athletes are also at an increased risk for sustaining injuries compared with their nonathlete peers,^31^ which may be conceptualized as significant stressors in and of themselves and are associated with an increased risk for depression.^7,35^ Taken together, it is evident that athletes face similar stressors as their nonathlete peers while also being subject to unique risk factors associated with their status as athletes.

While it is evident that student-athletes are susceptible to mental health-related difficulties and that injuries are associated with subsequent mental health concerns,^7,35^ the potential associations between these factors and the risk for injury are not well understood. The available literature examining the relationship between depression and anxiety and the risk for sports injury, broadly, is relatively mixed, with some research showing that anxiety and co-occurring depression/anxiety increased the risk for sports injury.^18^ In contrast, other research found that only depression was associated with an increased risk for injury while anxiety was protective against injury risk.^38^ Therefore, more work is needed in this domain. Further, it is currently unclear whether mental health concerns, like depression and anxiety, increase the risk of sustaining sports-related concussions. In other words, is the presence of depression and/or anxiety at baseline associated with increased vulnerability for concussion? One important theoretical model that can be used to conceptualize and frame this question of risk is the stress-injury model proposed by Williams and Andersen.^36^ The authors proposed that the severity of an athlete's stress response—influenced by personality factors, history of stressors, and coping resources—contributes to the increased risk for injury. Extrapolating to the present study, the presence of depression paired with trait anxiety and limited and/or ineffective coping resources may place athletes with co-occurring depression/anxiety at an inherently increased risk for injury compared with athletes without these symptoms. In line with this rationale, previous research has found that athletes reporting depression and anxiety are at an increased risk for sports-related injuries.^18^ However, 1 study examining pre- and postinjury risk factors for concussion found that depression and anxiety were not predictive of future concussion.^26^ This said, this study was limited in that it only examined depression and anxiety separately and did not examine the effects of co-occurring depression/anxiety. Given that some literature has shown that co-occurring depression/anxiety is associated with worse outcomes than depression or anxiety alone^11,16,25^ and athletes with co-occurring depression/anxiety tend to perform worse on neuropsychological testing and report more symptoms than athletes without affective symptoms,^33,34^ it is critical that research focuses on co-occurring affective symptoms. With these considerations in mind, the present study aimed to further explore whether depression and/or anxiety during preseason baseline assessment increased the likelihood that an athlete would sustain a future concussion during their collegiate career. Based on the available literature discussed above, as well as the mechanism of the increased likelihood of injury proposed in the stress-injury model, we hypothesized that (1) athletes reporting affective disturbance (e.g., depressive, anxiety, or co-occurring depressive/anxiety symptoms) at baseline would show an increased risk for prospective concussion compared with athletes without affective disturbance (i.e., healthy controls) and (2) athletes reporting co-occurring depressive/anxiety symptoms would be at a greater risk for prospective concussion compared with athletes experiencing either depressive symptoms or anxiety symptoms alone. This research is important from a translational standpoint, considering that concussions are one of the most common sports-related injuries.^15^ Notably, the median recovery time from concussion is around 13 days, with most athletes recovering within 1 month.^8^ This translates to a significant amount of time lost from sport, and athletes with affective and anxious/nervous symptomatology tend to experience delayed clinical recovery and take longer to return to play after sports-related concussion^12^; thus, their recovery times may be prolonged. This is important to understand and communicate to athletes, as there is likely a bidirectional relationship such that anxiety delays recovery and longer recovery/exacerbated symptoms lead to more anxiety. However, it is also important that we better understand whether there may be ways to reduce the risk of injury in the first place.

## Methods

### Participants

A total of 1051 collegiate athletes were involved in a concussion management program at our National Collegiate Athletic Association (NCAA) Division I university and received preseason (baseline) neuropsychological assessments between 2003 and 2019. All athletes were initially referred for baseline assessment by their athletic trainer or team physician as part of standard operating procedures when entering the athletic programs. The neuropsychological evaluation included a hybrid neuropsychological test battery, psychosocial questionnaires, and information about relevant concussion history. Athletes were only included in the present study if they completed baseline neuropsychological assessments—including the Beck Depression Inventory-Fast Screen (BDI-FS)^5^ and the NEO-Five Factor Inventory (NEO-FFI).^21^ Athletes who did not complete the neuropsychological evaluation, the BDI-FS, or the NEO-FFI were excluded from the analysis (n = 165). In addition, athletes evaluated for a nonsports-related concussion (eg, motor-vehicle accident while in-season) were also excluded from the analysis (n = 8). Therefore, the analyses of the present study included 878 (218 female) collegiate athletes. Athletes from the following sports underwent baseline assessment: football, men's and women's soccer, wrestling, men's and women's lacrosse, men's and women's ice hockey, men's and women's basketball, baseball, softball, crew, volleyball, and rugby. All data were collected before the COVID-19 pandemic.

### Procedures

The present study was conducted as part of the Sports Concussion Program at a large NCAA Division I university. The Sports Concussion Program is based on the “Sports as Laboratory Assessment Model.”^3,4^ As such, all participants were initially referred for baseline testing by their athletic trainer or team physician and completed a 2.5-hour comprehensive neuropsychological test battery as part of their standard care. Then, if athletes were diagnosed with a concussion by their athletic trainer or team physician during their collegiate athletic career, they were referred to our program for follow-up postconcussion evaluations. The neuropsychological test battery was administered by undergraduate research assistants or graduate students supervised by a PhD-level clinical neuropsychologist (P.A.A.). This study was conducted in compliance with the university's institutional review board (IRB) requirements and the American Psychological Association's ethical guidelines. All participants provided written informed consent, the study was approved by the behavioral committee of the IRB at our university, and the study was conducted in line with the Helsinki Declaration.

### Measures

A modified subscale of the NEO-FFI was used to measure symptoms of anxiety (NEO-FFI Anxiety Subscale). Possible scores ranged from 0 to 16, with higher scores indicating more severe anxiety. The rationale and support for developing this subscale can be found elsewhere, although the measure was normally distributed in our sample.^33^ Importantly, the NEO-FFI Anxiety Subscale has been shown to correlate with the Anxiety Sensitivity Index (ASI) at levels comparable to the ASI's correlation with other trait anxiety measures.^22,28,30,33^ NEO-FFI Anxiety subscale scores were dichotomized into 2 groups based on the presence of elevated anxiety symptomatology (≥10) or the absence of elevated anxiety symptomatology (<10), which is equivalent to cutoffs used in previous work.^33,34^ These cutoffs were consistent for both male and female patients.

The BDI-FS was used to measure depressive symptoms in this study. The BDI-FS consists of 7 items rated from 0 to 3, with higher scores indicating more severe depressive symptoms. The BDI-FS demonstrates value in measuring depressive symptoms in concussion populations because of its ability to discriminate between symptoms of a concussion and symptoms of depression by removing items related to neurovegetative symptoms that overlap with concussion symptoms.^29^ A large percentage of athletes reported a score of 0 on the BDI-FS, thus leading to a nonnormal, positive skewness in the present sample. As such, to maintain consistency with the NEO-FFI Anxiety Subscale and previous research, BDI-FS scores were dichotomized into 2 groups based on the presence of elevated depressive symptomatology (≥3) or the absence of elevated depressive symptomatology (<3). Similar to the NEO-FFI Anxiety Subscale, these cutoffs were consistent for both male and female patients, consistent with previous work.^33^

Athletes were then separated into 4 groups based on their anxiety and depressive symptom scores at baseline: healthy controls (n = 613; 132 [21.5%] women), depressive symptoms alone (n = 141; 35 [24.8%] women), anxiety symptoms alone (n = 56; 24 [42.9%] women), and co-occurring depressive/anxiety symptoms (n = 68; 27 [39.7%] women). Athletes without affective disturbance, as indicated by nonelevated depression and anxiety scores, were considered healthy controls. We analyzed variance to examine potential group differences; we found significant differences in terms of sex (*P* < .001) such that the anxiety symptoms alone and co-occurring depressive/anxiety groups consisted of significantly more female patients, although the groups did not differ in mean age (*P* = .40), estimated full-scale intelligence quotient (*P* = .07), or proportion of athletes reporting previous head injuries (*P* = .18). Additional descriptive data of participants are presented in Table 1. Importantly, rates of depressive and/or anxiety symptoms did not greatly differ throughout data collection (see Supplementary Materials).

**Table 1:** Participant Characteristics by Group^*a*^

Variable	Healthy Control	Depressive Symptoms Alone	Anxiety Symptoms Alone	Co-occurring Depressive/ Anxiety Symptoms	Total Sample
No. of athletes	613	141	56	68	878
% female	21.5	24.8	42.9	39.7	24.8
Age inyears, mean (SD)	18.52 (1.02)	18.57 (1.23)	18.30 (0.83)	18.44 (0.87)	18.50 (1.04)
% of athletes reporting previous concussion	32.8	40.6	27.3	39.1	34.2
Predicted FSIQ, mean (SD)	103.52 (5.94)	102.09 (7.04)	102.93 (5.41)	102.45 (6.70)	103.17 (6.17)
Race and ethnicity, n					
Asian	6	0	0	1	7
Black	110	41	7	12	170
Latinx	7	3	1	3	14
White	463	90	47	49	649
Multiracial	22	5	1	2	30
Other	5	2	0	1	8
Sport, n					
Baseball	1	0	0	1	2
Men’s basketball	38	9	1	4	64
Women’s basketball	21	21	1	9	40
Football	178	32	14	13	237
Men’s ice hockey	58	10	1	2	71
Women’s ice hockey	3	0	1	0	4
Men’s lacrosse	119	21	11	7	158
Women’s lacrosse	37	9	7	8	61
Women’s rugby	1	0	0	0	1
Softball	2	1	0	0	3
Men’s soccer	67	14	4	12	97
Women’s soccer	64	16	14	9	103
Women’s volleyball	1	0	0	0	1
Men’s wrestling	22	6	1	2	31
Other	1	2	1	1	4

a FSIQ, full-scale intelligence quotient.

### Statistical Analytic Plan

To address hypothesis 1, a logistic regression (enter method) was conducted with prospective concussion (yes/no) as the outcome variable and the affective group (healthy controls, depression alone, anxiety alone, and co-occurring depression/anxiety) as the predictor. Given that the predictor variable was categorical, we employed dummy coding, such that the healthy mood group was used as the reference group. Each of the 3 affective groups was individually compared with the healthy mood group. This approach allowed us to directly examine the change in the risk for concussion associated with affective disturbance compared with the healthy mood group. To address hypothesis 2, we conducted a series of chi-square analyses comparing the co-occurring depression/anxiety group to the depression alone and anxiety alone groups separately. This approach enabled us to examine the differential risk associated with co-occurring affective disturbance as opposed to either depression alone or anxiety alone. In terms of covariates, we conducted separate logistic regressions examining potential confounding variables. Some available research has found that headache^6^ and diagnosis of learning disorder or attention-deficit hyperactivity disorder^6,24^ increase the risk for injury; these factors were not significantly associated with the risk for concussion in our sample, so they were not included as covariates (all, *P* > .05). Previous research has also shown significant differences between sports in terms of risk for concussion.^9,13,14^ Consistent with this research, we found that sport significantly predicted concussion outcomes in our sample (*P* = .01); thus, sport was included as a covariate in the subsequent analyses. In addition, given that there were sex differences between affective groups in our sample and previous research has shown that female athletes sustain higher rates of concussion,^10^ sex was also examined as a potential covariate; however, sex was not significantly associated (*P* = .21) with the outcome measure (eg, prospective concussion) and was thus not included in the final regression model. Lastly, some available literature has found that the number of previous concussions an athlete has sustained increases the risk for future concussions,^6,32^ although other research has suggested that previous concussion and preinjury factors, largely, are not good predictors of future concussion.^17^ While there were no group differences in terms of athletes who sustained previous concussions, we examined the number of previous concussions as a potential confounder, given the lack of consensus in the available literature. We found that the number of previous concussions was not significantly associated (*P* = .25) with future concussions in our sample and was thus not included in the final regression model. All statistical analyses were conducted using R version 4.3.2 (R Core Team). An alpha value of 0.05 was used to determine statistical significance.

## Results

### Prevalence Rates of Concussion

**Figure 1.**
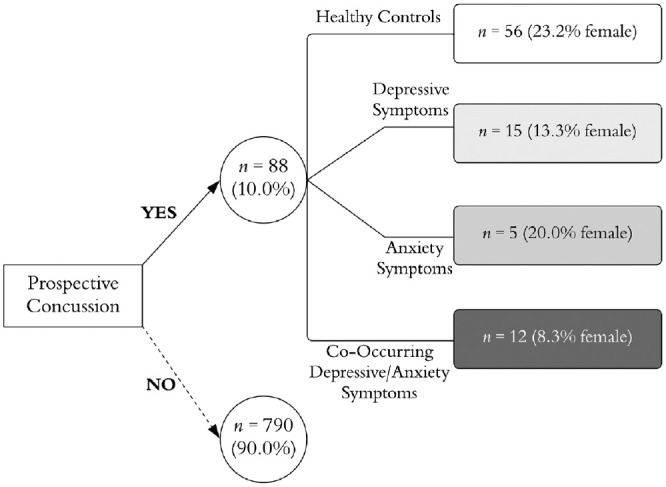
Longitudinal schematic of athletes classified by prospective concussion and affective group.

Of the 878 athletes who completed baseline testing, 88 (10%) athletes were diagnosed with a sports-related concussion during their collegiate career and referred for subsequent evaluation by their athletic trainer or team physician (Figure 1).

**Figure 2.**
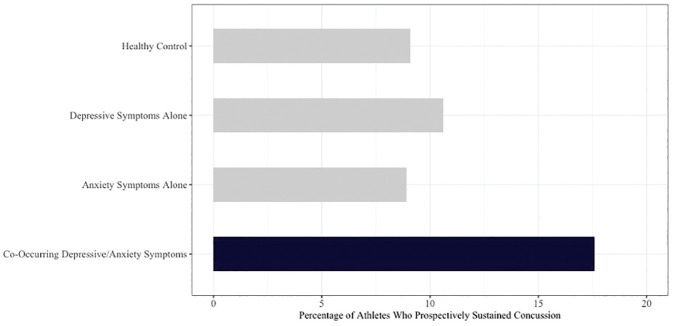
Prevalence rates of prospective concussion by the affective group. Percentages reflect the number of athletes within each separate baseline group (ie, healthy mood, depression alone, anxiety alone, or co-occurring depression/anxiety) who went on to sustain future concussions. For example, 17.6% (12 of 68) of the athletes in the baseline co-occurring depression/anxiety group sustained a future concussion.

There were no statistical differences between the percentage of male athletes (9.9%) versus female athletes (7.8%) who were referred for subsequent postconcussion testing. When examining prevalence rates by baseline group, we found that 9.1% of the healthy mood group sustained a prospective concussion compared with 10.6% of the depression alone group, 8.9% of the anxiety alone group, and 17.6% of the co-occurring depression/anxiety group (Figure 2).

Percentages reflect the number of athletes within each separate baseline group (ie, healthy mood, depression alone, anxiety alone, or co-occurring depression/anxiety) who went on to sustain future concussions. For example, 17.6% (12 of 68) of the athletes in the baseline co-occurring depression/anxiety group sustained a future concussion.

**Figure 3.**
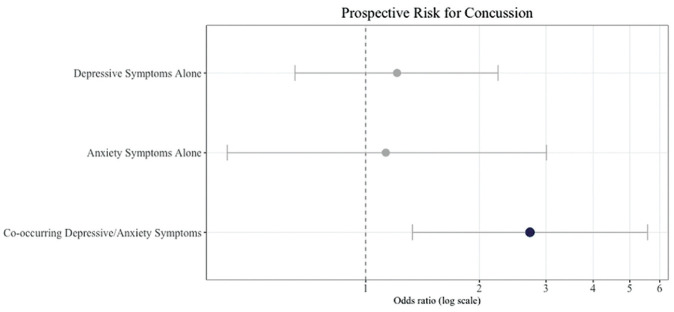
Odds for prospective concussion compared with athletes without affective disturbance. Logistic regression and odds ratios are compared with the healthy mood group as the reference. Therefore, the odds for prospective concussions are in relation to athletes without affective complaints.

Logistic regression analysis showed that, after controlling for sport, the co-occurring depression/anxiety group was more than twice as likely to be diagnosed with a prospective concussion compared with the healthy mood group (Figure 3 and Table 2). Neither the depressive symptoms alone nor the anxiety symptoms alone group was at significantly increased risk for future concussion compared with the healthy mood group (Table 2).

**Table 2:** Logistic Regression Results Comparing Affective Groups to the Healthy Mood Group on Prospective Risk for Concussion After Controlling for Sport^*a*^

Group Comparison	*B*	SE *B*	Wald	*P*	OR	95% CI
Healthy control vs depressive symptoms alone	0.19	0.32	0.36	.55	1.21	0.65-2.24
Healthy control vs anxiety symptoms alone	0.12	0.50	0.06	.81	1.13	0.43-3.01
Healthy control vs co-occurring depressive/anxiety symptoms	1	0.37	7.45	.01	2.72	1.33-5.57

a Degrees of freedom, 860. The statistical significance threshold was set at *P* < .05. OR, odds ratio.

Logistic regression and odds ratios are compared with the healthy mood group as the reference. Therefore, the odds for prospective concussions are in relation to athletes without affective disturbance.

Results of the chi-square analyses were consistent with the findings from the logistic regression in that athletes in the co-occurring depressive/anxiety symptoms group were significantly more likely to be diagnosed with prospective concussion compared with the healthy control group—χ^2^(1, *N* = 680) = 4.91 (odds ratio, 2.13; ϕ = .09; *P* = .03)—which implies more than doubled increased risk for future concussion for athletes with co-occurring depression/anxiety. However, there was no significant difference in the future diagnosis of a concussion between the co-occurring depressive/anxiety symptoms group and the depressive symptoms alone or anxiety symptoms alone groups (Table 3).

**Table 3:** Chi-square Comparisons Between Affective Groups^*a*^

Baseline Group	Concussion	No Concussion	χ^2^	*P*	φ	OR	95% CI
Co-occurring depressive/anxiety symptoms	12 (17.6)	56	—	—	—	—	—
Healthy control^*b*^	56 (9.1)	557	4.93	.03	.09	2.13	1.08-4.21
Depressive symptoms alone^*b*^	15 (10.6)	126	2.00	.19	.10	1.80	0.79-4.09
Anxiety symptoms alone^*b*^	5 (8.9)	51	1.97	.20	.13	2.19	0.72-6.63

a The statistical significance threshold was set at *P* < .05. Data are presented as n (%). OR, odds ratio. Dashes indicate data not available.

b Comparison is to the co-occurring depressive/anxiety symptoms group.

## Discussion

Given the high prevalence of depression and anxiety in college-aged populations, including collegiate athletes, this study aimed to examine whether baseline depression and anxiety increases the risk for prospective concussion. First and foremost, we found that athletes with co-occurring depressive/anxiety symptoms were more than twice as likely to be diagnosed with a prospective concussion compared with athletes without affective disturbance. This is similar to the findings of Li et al,^18^ who found a positive correlation between co-occurring depression/anxiety and the risk for sports injury more broadly. However, when examining depressive symptoms and anxiety symptoms separately, we found no significant differences between athletes with depressive symptoms alone and anxiety symptoms alone compared with athletes without affective disturbance, which is consistent with the findings of Putukian et al.^26^ Taken together, these findings suggest a unique risk associated with co-occurring depressive/anxiety symptoms and diagnosis of sports-related concussion. Providers should be aware of such risk, as it may be important to identify these athletes early (ie, during preseason assessments) to provide treatment and potentially reduce the risk of concussion. Second, researchers should be aware of this unique risk, as most of the available literature has tended to examine depression and anxiety separately. As such, it is critical that providers screen both depressive and anxiety symptoms and that future research continues to explore the effects of co-occurring depression and anxiety.

Notably, while these results indicate that the presence of co-occurring depression/anxiety places an athlete at an increased risk for injury, the specific mechanism is unclear. One possible explanation, in line with the stress and injury model proposed by Williams and Andersen,^36^ described above, is that some athletes may exhibit greater attentional and physiological responses to potentially stressful athletic situations because of personality traits, history of stressors, and poor coping skills, which may place them at greater risk for co-occurring depressive/anxiety symptoms as well as injury. This is supported by more recent neuropsychological research showing that athletes with co-occurring depression/anxiety showed poorer attention and slower processing speed compared with healthy controls,^33^ which may make it more difficult for these athletes to effectively attend to and/or process information efficiently during competition, which then places them at risk for injury. For example, one can imagine a scenario where an athlete is distracted or is having trouble devoting their entire attentional resources during their game or match and/or is slow to respond to events unfolding during competition, which then limits their capacity to quickly and efficiently protect themselves—whether from an opponent or by taking proprioceptive measures to reduce risk and severity of injury. A history of stressors—particularly negative life events—increases the risk for injury, as do high trait anxiety, elevated competitive state anxiety, and/or poor psychological coping skills. As such, athletes with co-occurring depressive/anxiety symptoms appear inherently at an increased risk for injury based on the theoretical stress and injury model. This is also in line with research showing an increased burden associated with multiple co-occurring conditions (eg, depression and anxiety) compared with only 1 condition.^12,29,30^ Another possible explanation is that athletes with depression and anxiety tend to report greater symptomatology on postconcussion symptom measures, even at baseline, compared with athletes with depression alone and anxiety alone^34^; thus, it is conceivable that these athletes are referred for postconcussion evaluation at a higher rate because of elevated symptomatology, which may or may not be related to concussion. This said, future work should more systematically evaluate potential mechanisms for an increased risk of injury associated with affective disturbance.

Overall, these findings demonstrate the significant risk associated with co-occurring depression/anxiety and highlight the importance of screening for mental health concerns at baseline. By screening for psychological symptoms at baseline, providers may be better equipped to quickly identify athletes who report mental health concerns and then provide treatment and/or recommendations. Not only is this important for athletes’ well-being but it may also help improve performance, considering mental health difficulties may impair athletic performance.^19^ Further, while there is no available research specifically examining the effectiveness of intervention for co-occurring depression/anxiety in reducing prospective risk for concussion, there is literature that shows that psychological intervention aimed at reducing anxiety and stress and improving coping skills (eg, cognitive-behavioral therapy and mindfulness-based interventions) broadly reduces the risk of injury. Tying in with the previously discussed stress and injury model, it is likely that reducing anxiety, processing/accepting negative life events, and improving coping skills through psychological intervention would translate to decreased stress response and, in turn, reduced risk for injury. Future work should specifically examine the effectiveness of these types of interventions for reducing symptoms of co-occurring depression/anxiety and concussion risk.

There are several limitations to the present study. First, the current sample consisted of relatively few athletes in the affective groups, which is largely a product of this study being naturalistic and the postconcussion groups being dependent upon injury (ie, nonmanipulatable event). As such, we were constrained by the general concussion rate and referral for postconcussion evaluation. Nevertheless, future work should continue to focus on longitudinal observation and expand upon these findings with more athletes who report depression and/or anxiety at baseline and then go on to sustain concussion. Regardless, we believe these findings are important in establishing that baseline affective disturbance may be a significant risk factor for future injury. Relatedly, it is important to note that the affective groups were determined based on self-reported symptomatology rather than formal clinical diagnosis. We believe that this approach captures a wider range of affective disturbance, especially considering that athletes tend to underreport affective symptoms^23^; nonetheless, future work should aim to include athletes with a formal diagnosis of mental health conditions and a previous history of mental health conditions. This study is also limited in that information regarding mental health treatment—including medications—between baseline and postconcussion evaluation was not available for all athletes. Relatedly, given that athletes were baseline tested at entry to athletics rather than annually, there are challenges associated with how mental health symptoms may or may not have changed over time. However, it is important to note that there were no athletes who reported co-occurring depressive/anxiety symptoms at baseline and then denied affective symptomatology at postconcussion. This underscores the importance of providing effective treatment to ameliorate these affective difficulties and potentially reduce the risk of poorer outcomes discussed in this study. We are also limited by the notable sex differences between groups. While we attempted to control for these differences by examining sex as a potential covariate—although it was not associated with our outcome of interest—future work would benefit from including more female athletes to have more representative groups and a more representative sample. This study is also limited in that our concussion management program is distinct from sports medicine. Hence, we depend on athletic trainers and/or sports medicine providers for referral for postconcussion evaluation. Therefore, it is possible that athletes tested at baseline were only evaluated by sports medicine providers and not referred for additional postconcussion evaluation. Further, several athletes who did not have available baseline testing were referred for postconcussion evaluation and were thus excluded from the study. It is also possible that athletes included in the study were monitored for different lengths of time (ie, first-year athletes versus fifth-year transfer student-athletes), which may introduce challenges when considering relative risk, especially if the athlete was not referred for subsequent testing. Another consideration pertaining to relative risk that was not available for all athletes but should be examined in future work is whether the athlete was a starter (or sport-equivalent), as this may introduce greater athletic exposure and, thus, greater risk for injury. As such, future work should more closely examine and, if appropriate, control for the number of athletic exposures over athletic careers. Last, for athletes who reported a history of previous concussion, information regarding the timing of the last injury was not collected; thus, there may be athletes experiencing symptoms associated with a previous injury during preseason evaluation; however, given that there were no group differences in terms of the number of athletes with previous concussion and that providers made referrals specifically for baseline assessments, it is believed that the results captured are an accurate reflection of the risk associated with depressive/anxiety symptomatology. Still, with all this in mind, other groups should aim to replicate these findings in different samples.

## Conclusion

Taken together, athletes with co-occurring depressive/anxiety symptoms at baseline showed a notably increased risk of being diagnosed with a future concussion, even after controlling for sport, compared with athletes without affective disturbance. This suggests that co-occurring depressive/anxiety symptoms confer a unique risk, which is associated with greater susceptibility to concussion diagnosis. As such, we recommend that providers screen for affective symptoms during individual preseason medical examinations and then refer athletes who screen positive for elevated depressive/anxiety symptoms to mental health professionals for further evaluation and treatment.

## Supplemental Material

sj-docx-1-ojs-10.1177_23259671241255932 – Supplemental material for Association Between Co-occurring Anxiety and Depressive Symptoms at Baseline and Risk for Sports-Related Concussion in Collegiate Athletes Supplemental material, sj-docx-1-ojs-10.1177_23259671241255932 for Association Between Co-occurring Anxiety and Depressive Symptoms at Baseline and Risk for Sports-Related Concussion in Collegiate Athletes by Garrett A. Thomas, Megan L. Bradson, Kaitlin E. Riegler, McKenna S. Sakamoto and Peter A. Arnett in Orthopaedic Journal of Sports Medicine

## References

[1] American College Health Association. American College Health Association-National College Health Assessment II: Reference Group Executive Summary Spring 2019. American College Health Association; 2019.

[2] Babiss LA Gangwisch JE. Sports participation as a protective factor against depression and suicidal ideation in adolescents as mediated by self-esteem and social support. J Dev Behav Pediatr. 2009;30(5):376. 19692930 10.1097/DBP.0b013e3181b33659

[3] Bailey CM Samples HL Broshek DK Freeman JR Barth JT. The relationship between psychological distress and baseline sports-related concussion testing. Clin J Sport Med. 2010;20(4):272-277. 20606512 10.1097/JSM.0b013e3181e8f8d8

[4] Barth JT Alves WM Ryan TV , et al. Mild head injury in sports: neuropsychological sequelae and recovery of function. In: Levin HS, Eisenberg HM and Benton AL, eds, Mild Head Injury. Oxford University Press; 1989:257-275.

[5] Beck A Steer R Brown G BDI-Fast Screen for Medical Patients Manual. San Antonio, TX: Psychological Corporation; 2000.

[6] Brett BL Kuhn AW Yengo-Kahn AM Solomon GS Zuckerman SL. Risk factors associated with sustaining a sport-related concussion: an initial synthesis study of 12,320 student-athletes. Arch Clin Neuropsychol. 2018;33(8):984-992. 29471410 10.1093/arclin/acy006

[7] Brewer BW Petrie TA. A comparison between injured and uninjured football players on selected psychosocial variables. Acad Athl J. 1995;10(Spring/1).

[8] Broglio SP McAllister T Katz BP , et al. The natural history of sport-related concussion in collegiate athletes: findings from the NCAA-DoD CARE Consortium. Sports Med. 2022;52(2):403-415. 34427877 10.1007/s40279-021-01541-7

[9] Chandran A Boltz AJ Morris SN , et al. Epidemiology of concussions in National Collegiate Athletic Association (NCAA) sports: 2014/15-2018/19. Am J Sports Med. 2022;50(2):526-536. 34898299 10.1177/03635465211060340

[10] Covassin T Moran R Elbin RJ. Sex differences in reported concussion injury rates and time loss from participation: an update of the National Collegiate Athletic Association Injury Surveillance Program from 2004-2005 through 2008-2009. J Athl Train. 2016;51(3):189-194. 26950073 10.4085/1062-6050-51.3.05 PMC4852524

[11] Dahm J Wong D Ponsford J. Validity of the depression anxiety stress scales in assessing depression and anxiety following traumatic brain injury. J Affect Disord. 2013;151(1):392-396. 23830002 10.1016/j.jad.2013.06.011

[12] D’Alonzo BA Bretzin AC Wiebe DJ , et al. The role of reported affective symptoms and anxiety in recovery trajectories after sport-related concussion. Am J Sports Med. 2022;50(8):2258-2270. 35647797 10.1177/03635465221098112 PMC10898515

[13] Daneshvar DH Nowinski CJ McKee A Cantu RC. The epidemiology of sport-related concussion. Clin Sports Med. 2011;30(1):1-17. 21074078 10.1016/j.csm.2010.08.006 PMC2987636

[14] Gessel LM Fields SK Collins CL Dick RW Comstock RD. Concussions among United States high school and collegiate athletes. J Athl Train. 2007;42(4):495-503. 18174937 PMC2140075

[15] Hootman JM Dick R Agel J. Epidemiology of collegiate injuries for 15 sports: summary and recommendations for injury prevention initiatives. J Athl Train. 2007;42(2):311-319. 17710181 PMC1941297

[16] LeMoult J Castonguay LG Joormann J McAleavey A. Depression. In: Castonguay LG Oltmanns TF , eds, Psychopathology: From Science to Clinical Practice. The Guilford Press; 2013:17-61.

[17] Lempke LB Schmidt JD Lynall RC. Athletic trainers’ concussion-assessment and concussion-management practices: an update. J Athl Train. 2020;55(1):17-26. 31855075 10.4085/1062-6050-322-18 PMC6961637

[18] Li H Moreland JJ Peek-Asa C Yang J. Preseason anxiety and depressive symptoms and prospective injury risk in collegiate athletes. Am J Sports Med. 2017;45(9):2148-2155. 28441037 10.1177/0363546517702847

[19] Lochbaum M Zanatta T Kirschling D May E. The profile of moods states and athletic performance: a meta-analysis of published studies. Eur J Investig Health Psychol Educ. 2021;11(1):50-70. 10.3390/ejihpe11010005 PMC8314345 34542449

[20] Locke B Wallace D Brunner J. Emerging issues and models in college mental health services. New Dir Stud Serv. 2016;2016(156):19-30.

[21] McCrae RR Costa PT. A contemplated revision of the NEO Five-Factor Inventory. Personal Individ Differ. 2004;36(3):587-596.

[22] McNally RJ Lorenz M. Anxiety sensitivity in agoraphobics. J Behav Ther Exp Psychiatry. 1987;18(1):3-11. 3558849 10.1016/0005-7916(87)90065-6

[23] Meier TB Brummel BJ Singh R Nerio CJ Polanski DW Bellgowan PSF . The underreporting of self-reported symptoms following sports-related concussion. J Sci Med Sport. 2015;18(5):507-511. 25150463 10.1016/j.jsams.2014.07.008

[24] Nelson LD Pfaller AY Rein LE McCrea MA. Rates and predictors of invalid baseline test performance in high school and collegiate athletes for 3 computerized neurocognitive tests: ANAM, Axon sports, and ImPACT. Am J Sports Med. 2015;43(8):2018-2026. 26059178 10.1177/0363546515587714 PMC4747101

[25] Pratt LA Druss BG Manderscheid RW Walker ER. Excess mortality due to depression and anxiety in the United States: results from a nationally representative survey. Gen Hosp Psychiatry. 2016;39:39-45. 26791259 10.1016/j.genhosppsych.2015.12.003 PMC5113020

[26] Putukian M Riegler K Amalfe S Bruce J Echemendia R. Preinjury and postinjury factors that predict sports-related concussion and clinical recovery time. Clin J Sport Med. 2021;31(1):15-22. 30540572 10.1097/JSM.0000000000000705

[27] Reardon CL Factor RM. Sport Psychiatry. Sports Med. 2010;40(11):961-980. 20942511 10.2165/11536580-000000000-00000

[28] Reiss S Peterson RA Gursky DM McNally RJ. Anxiety sensitivity, anxiety frequency and the prediction of fearfulness. Behav Res Ther. 1986;24(1):1-8. 3947307 10.1016/0005-7967(86)90143-9

[29] Riegler KE Guty ET Arnett PA. Validity of the ImPACT Post-Concussion Symptom Scale (PCSS) affective symptom cluster as a screener for depression in collegiate athletes. Arch Clin Neuropsychol. 2019;34(4):563-574. 30418516 10.1093/arclin/acy081

[30] Sandin B Chorot P McNally RJ. Anxiety Sensitivity Index: normative data and its differentiation from trait anxiety. Behav Res Ther. 2001;39(2):213-219. 11153974 10.1016/s0005-7967(00)00009-7

[31] Simon JE Docherty CL. Current health-related quality of life is lower in former Division I collegiate athletes than in non-collegiate athletes. Am J Sports Med. 2014;42(2):423-429. 24318608 10.1177/0363546513510393

[32] Teel EF Marshall SW Shankar V McCrea M Guskiewicz KM. Predicting recovery patterns after sport-related concussion. J Athl Train. 2017;52(3):288-298. 28387552 10.4085/1062-6050-52.1.12 PMC5384825

[33] Thomas GA Guty ET Riegler KE Arnett PA. Comorbid affective symptomatology and neurocognitive performance in college athletes. J Int Neuropsychol Soc. 2022;28(2):177-187. 33949296 10.1017/S1355617721000412

[34] Thomas GA Riegler KE Guty ET Arnett PA. Relationship between self-reported concomitant depressive and anxiety symptoms and the Post-Concussion Symptoms Scale (PCSS). J Int Neuropsychol Soc. 2022;28(10):1064-1074. 34895377 10.1017/S135561772100134X

[35] Vargas G Rabinowitz A Meyer J Arnett PA. Predictors and prevalence of postconcussion depression symptoms in collegiate athletes. J Athl Train. 2015;50(3):250-255. 25643158 10.4085/1062-6050-50.3.02 PMC4477919

[36] Williams JM Andersen MB. Psychosocial antecedents of sport injury: review and critique of the stress and injury model. J Appl Sport Psychol. 1998;10(1):5-25.

[37] Wolanin A Gross M Hong E. Depression in athletes: prevalence and risk factors. Curr Sports Med Rep. 2015;14(1):56. 25574886 10.1249/JSR.0000000000000123

[38] Yang J Cheng G Zhang Y Covassin T Heiden EO Peek-Asa C. Influence of symptoms of depression and anxiety on injury hazard among collegiate American football players. Res Sports Med. 2014;22(2):147-160. 24650335 10.1080/15438627.2014.881818

