# Supplementary material for: Association Between Co-occurring Anxiety and Depressive Symptoms at Baseline and Risk for Sports-Related Concussion in Collegiate Athletes
Source: Orthop J Sports Med. 2024 Jun 19;12(6):23259671241255932. doi: 10.1177/23259671241255932 (PMC11191608; doi:10.1177/23259671241255932)
Supplement: sj-docx-1-ojs-10.1177_23259671241255932 – Supplemental material for Association Between Co-occurring Anxiety and Depressive Symptoms at Baseline and Risk for Sports-Related Concussion in Collegiate Athletes [file sj-docx-1-ojs-10.1177_23259671241255932.docx]

**Supplementary Table 1:** Rates of Affective Complaints by Year

| **Year** | Healthy Control | Depressive Symptoms | Anxiety Symptoms | Co-Occurring Depressive/Anxiety Symptoms | Total |
| --- | --- | --- | --- | --- | --- |
| 2003 | 1 (50.0%) | --- | 1 (50.0%) | --- | 2 |
| 2004 | 38 (64.4%) | 11 (18.6%) | 4 (6.8%) | 6 (10.2%) | 59 |
| 2005 | 61 (60.4%) | 21 (20.8%) | 8 (7.9%) | 11 (10.4%) | 101 |
| 2006 | 56 (69.1%) | 14 (17.3%) | 2 (2.5%) | 9 (11.1%) | 81 |
| 2007 | 64 (68.1%) | 16 (17.0%) | 5 (5.3%) | 9 (9.6%) | 94 |
| 2008 | 54 (68.4%) | 11 (13.9%) | 8 (10.1%) | 6 (7.6%) | 79 |
| 2009 | 51 (71.8%) | 11 (15.5%) | 6 (8.5%) | 3 (4.2%) | 71 |
| 2010 | 55 (76.4%) | 9 (12.5%) | 5 (6.9%) | 3 (4.2%) | 71 |
| 2011 | 60 (77.9%) | 8 (10.4%) | 6 (7.8%) | 3 (3.8%) | 77 |
| 2012 | 71 (78.0%) | 12 (13.2%) | 4 (4.4%) | 4 (4.4%) | 91 |
| 2013 | 36 (69.2%) | 7 (13.5%) | 3 (5.8%) | 6 (11.5%) | 52 |
| 2014 | 30 (61.2%) | 15 (30.6%) | 2 (4.1%) | 2 (4.1%) | 48 |
| 2015 | 18 (72.0%) | 2 (8.0%) | 2 (8.0%) | 3 (12.0%) | 25 |
| 2016 | 7 (77.8%) | 1 (11.1%) | 1 (11.1%) | --- | 9 |
| 2017 | 5 (45.5%) | 3 (27.3%) | 1 (9.1%) | 2 (18.2%) | 11 |
| 2018 | 5 (62.5%) | 2 (25.0%) | --- | 1 (12.5%) | 8 |
| 2019 | 1 (100.0% | --- | --- | --- | 1 |
